# A Silicon-Based Field-Effect Biosensor for Drug-Induced Cardiac Extracellular Calcium Ion Change Detection

**DOI:** 10.3390/bios14010016

**Published:** 2023-12-28

**Authors:** Yong Qiu, Chiyu Ma, Nan Jiang, Deming Jiang, Zhengyin Yu, Xin Liu, Yuxuan Zhu, Weijie Yu, Fengheng Li, Hao Wan, Ping Wang

**Affiliations:** 1 Biosensor National Special Laboratory, Key Laboratory for Biomedical Engineering of Education Ministry, Department of Biomedical Engineering, Zhejiang University, Hangzhou 310027, China; zjubme_qy@zju.edu.cn (Y.Q.); mcy1996@zju.edu.cn (C.M.); 21915031@zju.edu.cn (N.J.); 21618488@zju.edu.cn (D.J.); liuxin8234@zju.edu.cn (X.L.); yuxuanzhu@zju.edu.cn (Y.Z.); yuweijie@zju.edu.cn (W.Y.); 22115006@zju.edu.cn (F.L.); 2 The MOE Frontier Science Center for Brain Science & Brain-Machine Integration, Zhejiang University, Hangzhou 310027, China; 3 State Key Laboratory of Transducer Technology, Chinese Academy of Sciences, Shanghai 200050, China; yuzy@mail.sim.ac.cn

**Keywords:** field-effect biosensors, microelectrode array, extracellular calcium ion, extracellular field potentials, L-type calcium channel, cardiovascular drug screening

## Abstract

Calcium ions participate in the regulation of almost all biological functions of the body, especially in cardiac excitation–contraction coupling, acting as vital signaling through ion channels. Various cardiovascular drugs exert their effects via affecting the ion channels on the cell membrane. The current strategies for calcium ion monitoring are mainly based on fluorescent probes, which are commonly used for intracellular calcium ion detection (calcium imaging) and cannot achieve long-term monitoring. In this work, an all-solid-state silicone–rubber ion-sensitive membrane was fabricated on light-addressable potentiometric sensors to establish a program-controlled field-effect-based ion-sensitive light-addressable potentiometric sensor (LAPS) platform for extracellular calcium ion detection. L-type calcium channels blocker verapamil and calcium channel agonist BayK8644 were chosen to explore the effect of ion channel drugs on extracellular calcium ion concentration in HL-1 cell lines. Simultaneously, microelectrode array (MEA) chips were employed to probe the HL-1 extracellular field potential (EFP) signals. The Ca^2+^ concentration and EFP parameters were studied to comprehensively evaluate the efficacy of cardiovascular drugs. This platform provides more dimensional information on cardiovascular drug efficacy that can be utilized for accurate drug screening.

## 1. Introduction

Calcium ions are indispensable for various physiological activities and are of great significance in maintaining the homeostasis of an organism’s environment. They are involved in regulating almost all biological functions of the organism, including heart and muscle contraction, neural information transmission, learning and memory, embryonic formation and development, cell proliferation and apoptosis, cell division and differentiation, cell energy metabolism, protein phosphorylation and dephosphorylation modification, gene expression and regulation, and so on [1,2]. Various pharmaceuticals also exert their effects by affecting specific proteins on the cell membrane that transport Ca^2+^. Studies have shown that L-type calcium channel inhibitors can be used to treat hypertension, and the N-type calcium channel blocker ziconotide can be used to treat severe and chronic pain [3]. In the tumor environment, many of the calcium-transport proteins intersect directly with processes that are important in cancer progression [4]. Although we know that it exerts its effects at numerous locations inside and outside cells, the knowledge of intracellular Ca^2+^ signaling vastly eclipses what is known about extracellular Ca^2+^ ‘signaling’ [5]. Therefore, it is crucial to detect extracellular calcium ions to understand how and when Ca^2+^ might change in the local microenvironment outside of cells.

The calcium ion detection kit is an endpoint method and cannot meet the needs of long-term monitoring. Laboratory analytical instruments with ultra-high detection accuracy have the drawbacks of costly equipment and cumbersome operation. Potentiometric sensors that feature small size and low cost, including ion-selective electrodes (ISEs) [6,7,8,9], ion-sensitive field-effect transistors (ISFETs) [10,11,12,13,14,15], and light-addressable potentiometric sensors (LAPSs) [16,17,18,19,20], have been widely used in ion detection.

As a type of field-effect device, compared to a field-effect transistor (FET), LAPS devices with electrolyte-insulator-semiconductor (EIS) structures have advantages including a simple device structure, the ease of modifying sensitive materials, and flexibility in defining detection areas. The insulator material (usually SiO_2_, Si_3_N_4_, and Al_3_O_4_) of LAPS chips has good biocompatibility and a small sample size requirement and has been successfully applied in the metabolic detection of cells [16,17,21,22,23,24,25] and bacteria [26,27,28]. However, the applications of LAPS biosensors only include metabolic pH monitoring in a solution environment. Therefore, it is necessary to expand the detection function of LAPS for other ions.

**Figure 1:**
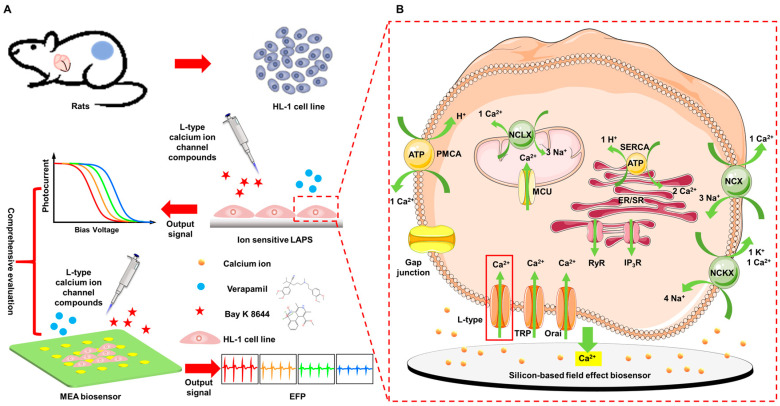
Schematic diagram of the calcium-ion-sensitive LAPS platform combined with an MEA chip for comprehensive evaluation of the efficacy of ion channel drugs; (**A**) schematic diagram of the efficacy of a comprehensive evaluation based on calcium-ion-sensitive LAPS chips and MEA chips; (**B**) schematic diagram of the main channels and transporters for extracellular/intracellular Ca^2+^ cycling; L-type Ca^2+^ channel, one of the voltage-gated Ca^2+^ (Cav) channels; TRP, transient receptor potential (TRP) family; ORA1, the calcium-release-activated calcium channel protein 1; MCU, mitochondrial calcium uniporter; NCLX, the mitochondrial Na^+^/Ca^2+^ exchanger; PMCA, plasma membrane Ca^2+^-transporting ATPase; NCX, Na^+^-Ca^2+^ exchanger; NCKX, Na^+^-Ca^2+^-K^+^ exchanger; SERCA, sarcoplasmic/ER Ca^2+^-ATPases; IP_3_R, inositol 1,4,5-trisphosphate receptor; RyR, ryanodine receptor.

In this work, an all-solid-state silicone-rubber ion-sensitive membrane was fabricated on partially thinned silicon wafers as light-addressable potentiometric sensors (LAPSs) to establish a program-controlled field-effect-based ion-sensitive LAPS platform for extracellular calcium ion detection (Figure 1A). The L-type calcium channel blocker verapamil and the calcium channel agonist Bayk8644 (the blue circle and red pentagram in Figure 1A, respectively) were chosen to explore the effect of ion channel drugs on extracellular calcium ion concentration in the HL-1 cell lines. Simultaneously, microelectrode array (MEA) chips were employed to record the HL-1 extracellular field potential (EFP) signals. The extracellular Ca^2+^ concentration and EFP parameters were studied to achieve a comprehensive evaluation of the efficacy of cardiovascular drugs. This platform provides more dimensional information on calcium ions that can be utilized for accurate drug screening.

## 2. Materials and Methods

### 2.1. Principle

#### 2.1.1. Biological Basis of Extracellular/Intracellular Ca^2+^ Cycling

The level of cytoplasmic resting free Ca^2+^ in mammalian cells is generally controlled at 100–200 nM, while the free Ca^2+^ in the extracellular fluid is maintained at the level of mM. The steep but strictly controlled concentration gradient of calcium ions inside and outside the cell membrane, as well as between the cytoplasm and organelles, is maintained and dynamically regulated according to the needs of the cell, relying on the instruments of Ca^2+^ homeostasis—the collaborative work of various ion channels, ion pumps, and transporters [1,2,29,30]. Figure 1B illustrates the main Ca^2+^ channels, pumps, and exchangers of the plasma membrane, and intracellular organelles.

There are three major pathways for Ca^2+^ entrance across the plasma membrane. They are categorized into voltage-gated Ca^2+^ (Cav) channels (subdivided into L-type, T-type, P/Q-type, R-type, and N-type; an example of an L-type channel is shown) [31], the transient receptor potential (TRP) family [32], and the calcium-release-activated calcium channel protein 1 (ORAI1) [33]. Orai is a unique type of Ca^2+^ channel involved in a mechanism known as store-operated calcium entry (SOCE), which is sensed by the stromal interaction molecule 1 (STIM1). STIM1 proteins are redistributed and interact with ORAI1 proteins, allowing extracellular Ca^2+^ to enter the plasma membrane, refilling the store when endoplasmic reticulum (ER) or sarcoplasmic reticulum (SR) Ca^2+^ stores are exhausted.

There are two main ligand-gated channels that mediate Ca^2+^ store release from ER/SR to intracellular fluid. They are the inositol 1,4,5-trisphosphate receptor (IP_3_R) and the ryanodine receptor (RyR). The calcium-induced calcium release (CICR) process is activated by RyR. In cardiomyocytes, CICR is triggered by the Ca^2+^ influx across the plasma membrane through Cav channels. It provides the majority of Ca^2+^ ions that support contraction [34,35]. Other intracellular organelles have Ca^2+^-transporting proteins, such as the mitochondrial calcium uniporter (MCU) complex, and the mitochondrial Na^+^/Ca^2+^ exchanger (NCLX) [36].

There are one pump and two transporters to move Ca^2+^ out of the cell: the plasma membrane Ca^2+^-transporting ATPase (PMCA) pump, Na^+^-Ca^2+^ exchanger (NCX), and the Na^+^-Ca^2+^-K^+^ exchanger (NCKX). PMCA exhibits high affinity but low capacity, while the exchangers exhibit low affinity but high capacity [34]. Through the synergistic effect of these channels, pumps, and transporters, calcium homeostasis is maintained together under such conditions in which the level of free Ca^2+^ in the extracellular fluid is much higher than the level of cytosol.

#### 2.1.2. The Principle of Calcium Ion Detection Based on Ion-Sensitive LAPS

**Figure 2:**
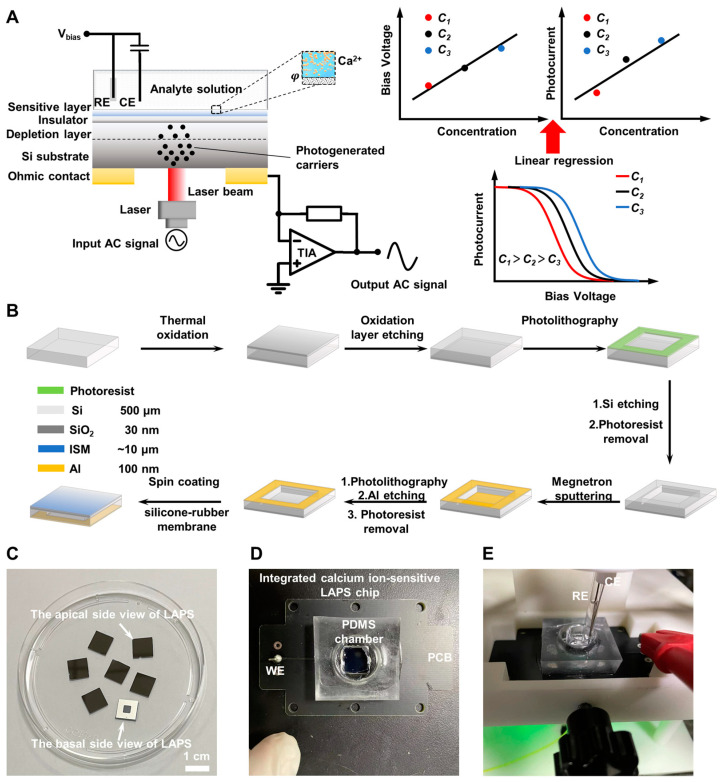
The detection principle and fabrication of the calcium-ion-sensitive LAPS chips; (**A**) principle of calcium ion detection by the calcium-ion-sensitive LAPS chips; (**B**) fabrication processing flow of the calcium ion-sensitive LAPS chips; (**C**) photograph of the LAPS chips (scale bar = 1 cm); (**D**) photograph of the PDMS cell chamber and PCB integrated with the calcium-ion-sensitive LAPS chip; (**E**) photograph of the calcium-ion-sensitive LAPS chip with Ag/AgCl reference electrode (RE) and Pt wire counter electrode (CE) mounted on the 3D printed scaffold.

A schematic of the calcium ion-sensitive LAPS structure is shown in Figure 2A. Doped silicon serves as the semiconductor substrate material, and the metal layer is deposited on the silicon substrate to form ohmic contact, connecting peripheral circuits. A thin SiO_2_ layer is used as the insulating layer, and a silicone-rubber ion-sensitive membrane modified on the surface of the insulating layer is used as the Ca^2+^-sensitive material. At the same time, a DC bias voltage is applied on the reference electrode (RE) of the LAPS system, and a depletion region is formed at the insulation/semiconductor interface. After illuminating a frequency-modulated light at the top/bottom of the sensor, hole–electron pairs are generated and continue to diffuse to the depletion region, resulting in an AC photocurrent with the same frequency [37]. The ohmic contact on the sensor substrate is grounded through a trans-impedance amplifier (TIA) to convert the photocurrent signal into a voltage signal for acquisition. The response mechanism of calcium ion-sensitive LAPS can be found in Figure S1.

When the sensitive layer contacts the target solution, Ca^2+^ enters into the ion-sensitive membrane and combines with the ionophore due to the concentration gradient [38]. Then, the surface potential of the LAPS changes, resulting in a change in depletion region thickness [17], which can be obtained via the value of the AC signal generated by the LAPS. After applying a linearly varying bias voltage, a series of photocurrent values can be obtained, and the photocurrent–bias voltage characteristic curve (I–V characteristic curve) can be established.

The bias voltage value of the inflection point (the point where the second derivative is zero) on the characteristic curve is calculated and usually selected as the working point of the LAPS in actual measurement [21]. Because the sensor is the most sensitive to analyte changes at the working point, its detection sensitivity is the highest.

As the surface potential of the LAPS changes with the concentration of the analyte, the I–V characteristic curve also shifts accordingly. This change can be quantified by measuring the horizontal/vertical displacement of the working point, obtaining the standard curve between the voltage/current at the working point and the concentration of the analyte. By bringing the change in phase boundary potential into the standard curve, the concentration change of the measured substance in the solution can be calculated, thus achieving the purpose of quantitative measurement. The detection area is determined using the illumination area, and the entire chip can be divided into multiple array detection areas, with specific areas fixed for Ca^2+^-sensitive materials.

The definition of the pH value is the concentration index of hydrogen ions in the electrolyte, expressed by the negative logarithmic formula of hydrogen ion concentration. Analogous to the definition of pH, we define pCa as the negative logarithm of calcium ion concentration.

### 2.2. Materials and Reagents

We purchased 4-inch N-type silicon wafers (500 μm thick, <100>, 1~10 Ω·cm) from Hangzhou Lijing Technology, Hangzhou, Zhejiang province, China. Silicone rubber RTV 730 was purchased from Dow Corning. N,N-dicyclohexyl-N0, N′-dioctadecyl-3-oxapentanediamide (ETH 5234, calcium ionophore IV), potassium tetrakis (3,5-bis(trifluoromethyl)phenyl) borate (KTFPB), sodium tetrakis (3,5-bis(trifluoromethyl)phenyl) borate (NaBARF), and tetrahydrofuran (THF) were purchased from Sigma-Aldrich. The conducting polymer poly(3-octyl-thiophene) (P3OT) and Dulbecco’s modified Eagle medium (DMEM) were purchased from Thermo Fisher. Analytically pure NaCl, KCl, CaCl_2_, MgCl_2_, and CH_3_COOLi powders were purchased from China National Pharmaceutical Group Chemical Reagent Co., Ltd. Aqueous solutions were prepared with deionized water (>18 MΩ·cm, Milli Q).

### 2.3. Calcium-Ion-Sensitive LAPS Chip Fabrication

The procedures for LAPS fabrication are demonstrated in Figure 2B. The n <100> silicon wafers (4 inches, 500 μm thick, 1~10 Ω·cm) were subjected to thermal oxidation, and a 30 nm thick SiO_2_ layer was grown on both sides. Then, the SiO_2_ layer from the rough surface was removed using 5% HF solution, and the central part of the Si substrate was thinned to 100 μm via photolithography and silicon etching. The reason why 100 μm thick silicon wafers were not directly used is that they cannot provide sufficient mechanical strength and are prone to fragmentation. After cleaning with deionized water and drying with nitrogen, a 100 nm thick aluminum layer was magnetron-sputtered on the basal area of the LAPS, and the metal layer of the silicon thinning area was removed via photolithography and Al etching, forming an ohmic contact. Finally, the wafer was sliced into 1 cm × 1 cm chips, with a thinning area of 4 mm × 4 mm in the center of the back, and then washed sequentially with acetone, ethanol, and deionized water for further use (see Figure 2C for a photograph of the LAPS chips (both apical and basal side views)).

For preparation of the calcium-ion-sensitive membrane solution, 300 mg of RTV 730 silicone rubber was weighed and dissolved in 1.5 mL of THF in a 10 min ultrasonic water bath. Then, the mixture was centrifuged at 6000 rpm for 20 min to remove adhesive components that affect optimal functionality [39,40]. After centrifugation, 1.4 mL of supernatant was pipetted and mixed with 3.61 mg of calcium ionophore IV and 1.33 mg of KTFPB. Finally, a 30 min ultrasonic water bath was used to fully dissolve the solute to obtain the membrane mixture.

Before modifying the calcium-ion-sensitive membrane, it was necessary to pre-modify a layer of P3OT on the SiO_2_ layer of the LAPS chips to suppress the formation of an aqueous layer between the ISM and solid-state contact. The chip was adsorbed onto the rotary table of the homogenizer via the negative pressure of the vacuum pump. A 50 μL 25 mM P3OT-THF solution was pipetted and dropped onto the center of the chip, and the spin coat was executed at 500 rpm for 30 s. After drying for 5 min, a 60 μL membrane mixture solution was spin-coated on the P3OT layer with the same parameters and sat overnight at room temperature. After that, the chip was immersed in 10^−2^ M Ca^2+^ solution for 2 h to improve membrane uniformity and maintain activity [41]. The Ca^2+^-sensitive LAPS chip was bonded to the solder pads of printed circuit boards (PCBs) with conductive silver adhesive to achieve connection with peripheral circuits, and the PDMS chamber was bonded with epoxy resin adhesive as the detection chamber (Figure 2D). The integrated chip was used for subsequent experiments. The Ag/AgCl reference electrode (RE), Pt wire counter electrode (CE), and the LAPS chip working electrode (WE) were mounted on the 3D-printed scaffold (Figure 2E).

### 2.4. Detection System Set Up of Ca^2+^-Sensitive LAPS

**Figure 3:**
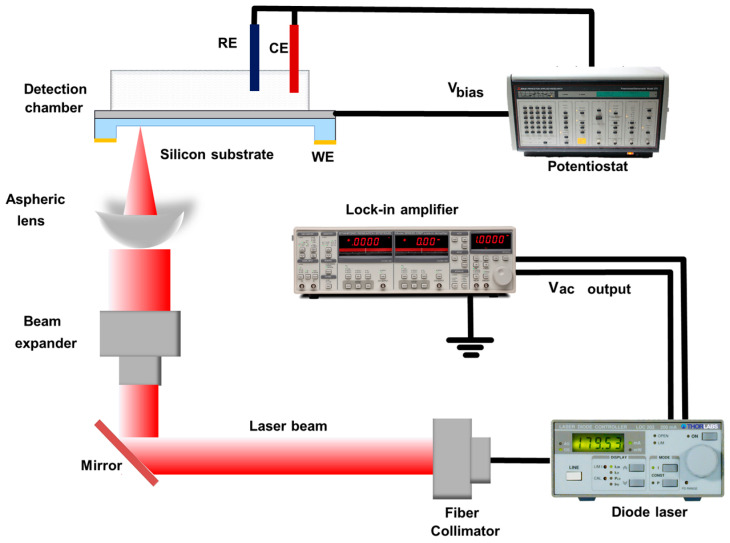
Schematic of the Ca^2+^-sensitive LAPS detection system setup.

Figure 3 describes the schematic of the Ca^2+^-sensitive LAPS detection system. The pigtailed laser diode with single mode fiber (LP405C1, Thorlabs, Newton, NJ, USA) was driven by a laser diode driver (LDC202C, Thorlabs, Newton, NJ, USA) under 15 mW and modulated by a lock-in amplifier (SR830, Stanford Research System, Sunnyvale, CA, USA) with a 10 kHz sinusoidal modulation signal. The modulated laser was firstly collimated using a fiber collimator, which was mounted in a homemade holder on the optical platform, then reflected via a 45° optical mirror and expanded by a beam expander. The laser beam was finally focused via an aspheric lens on the thinned area of the Ca^2+^-sensitive LAPS chip as laser excitation source. The Ag/AgCl reference electrode (RE), Pt wire counter electrode (CE), and the LAPS chip working electrode (WE) connected through the ohmic contact were adopted to jointly form an electrochemical three-electrode system. The constant bias voltage and the I–V-converting were implemented via a potentiostat (Model 273A, EG&G, San Diego, CA, USA). After I–V converting and lock-in amplification, 10 kHz AC voltage could be detected, corresponding to hole–electron pairs; creation by the radiation absorption from the laser.

### 2.5. Cell Culture

The preparation of the HL-1 cardiomyocyte culture medium included Claycomb medium (51800C, Sigma Aldrich, Darmstadt, Germany), 10% fetal bovine serum (F2442, Sigma Aldrich, Darmstadt, Germany), 1% norepinephrine (10 mM stock solution), 1% L-glutamine (200 mM stock solution, Gibco, Waltham, MA, USA), and 1% penicillin/streptomycin (penicillin: 100 U/mL; streptomycin: 100 μg/mL). Due to the presence of photosensitive components in the culture medium, the prepared culture medium needed to be filtered and sterilized and protected from light.

Before cell culture, the T25 culture flask was coated with gelatin/fibronectin mixture solution. The coating solution was dissolved in 0.5% fibronectin solution (1 mg/mL) in the pre-prepared gelatin solution (0.02%), and the final concentration of fibronectin was 5 μg/mL. The coating solution was filtered and sterilized, and stored at 4 °C. The HL-1 cardiomyocyte was cultured inside a humidified incubator at 37 °C and 5% CO_2_. When the growth state of HL-1 cardiomyocyte in the culture flask reached 100% fusion, special trypsin EDTA (T3924, Sigma Aldrich) digestive solution was used for digestion, and the cells were passaged at a 1:3 split ratio.

## 3. Results and Discussion

### 3.1. Ca^2+^-Sensitive LAPS Chip Calibration

**Figure 4:**
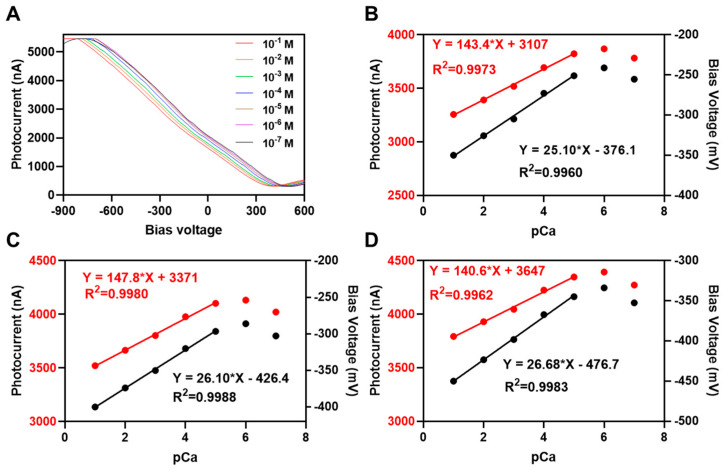
Calibration results of Ca^2+^-ensitive LAPS chips: (**A**) series of photocurrent–bias voltage (I–V) curves of the calcium-ion-sensitive LAPS chips with the corresponding concentration gradient CaCl_2_ solutions (10^−7^ M~10^−1^ M), expressed as the average of three repeated measurements; (**B**–**D**) the fitting curves under constant current and constant voltage measurement mode of three separate sensor chips (voltage sensitivity and current sensitivity are represented in black and red, respectively). The solid lines represent the calibration curve fitted to the working point within the linear working area of the sensor, and the points refer to the working point within the non-linear working area of the sensor.

The sensitivity of the Ca^2+^-sensitive LAPS chip was calibrated with a series of concentration gradient CaCl_2_ solutions (10^−7^ M~10^−1^ M). Referring to the proposed equivalent circuit model of a LAPS [42], the series impedance of the LAPS system is composed of solution resistance, double-layer capacitance, semiconductor resistance, ohmic contact resistance, and external circuit impedance. Due to the significant influence of the solution resistance component on the current signal amplitude, it is necessary to ensure a certain amount of supporting electrolyte in the analyte solution (0.1 M lithium acetate was used in this work). The series of photocurrent–bias voltage characteristic curves measured under corresponding concentration solutions are shown in Figure 4A, and each curve is the average of three repeated measurements. We found that the I−V curves shifted from left to right as the Ca^2+^ concentration decreased. In the concentration range of 10^−1^~10^−6^ M, the I−V curves can be clearly distinguished from each other, but the curve of 10^−7^ M overlaps with the curves of 10^−5^~10^−6^ M and cannot be distinguished, indicating that the concentration of 10^−7^ M exceeded the linear detection range of the sensor.

To further quantitatively analyze the limit of detection (LOD) and linear detection range of sensors, the bias voltage/photocurrent–pCa linear regression of three separate calcium-sensitive LAPS chips was carried out. Figure 4B–D show fitting curves under constant current measurement mode and constant voltage measurement mode. The fitting curves had a high correlation (R^2^ > 0.99) with both the bias voltage and the photocurrent. The voltage sensitivity results were 25.10, 26.10, and 26.68 mV/pCa, which are close to the Nikolsky equation theoretical values (the divalent cation is 29.6 mV/p*). The current sensitivity results were 143.4, 147.8, and 140.6 nA/pCa. In accordance with the IUPAC recommendations [43], the LOD of ion-selective electrodes could be obtained from the concentration value corresponding to the intersection of the extension of the fitting lines of high- and low-sensitivity areas. In constant current measurement mode, the calculated LODs of these three sensor chips were 2.50 × 10^6^, 2.40 × 10^−6^, and 2.39 × 10^−6^ M. In constant voltage measurement mode, the LODs were 2.66 × 10^−6^, 3.01 × 10^−6^, and 2.34 × 10^−6^ M. The method for calculating the limit of detection (LOD) can be found in Figure S2 [43].

It is shown that both the constant current and voltage measurement modes had nearly the same LOD, but the constant voltage measurement mode was more sensitive due to the photocurrent amplification effect, which is suitable for detecting the small Ca^2+^ concentration changes in the extracellular microenvironment.

For the investigation of response time and long-time stability, the response time (t_99_) can be seen in Figure S3A and was calculated to be 4.6 s during the constant-voltage mode measuremen, the long-time stability results showed a standard deviation of ±18.338 nA (Figure S3B) during a measurement period of 180 min. One explanation for the current in the cutoff zone is not strictly zero [44,45,46] in Figure 4A can be found in Supplementary Material in Figure S4.

### 3.2. Characterization of Potentiometric Selectivity Coefficient

In the detection of calcium ions in the extracellular microenvironment, the culture medium solution environment is complex, including not only some inorganic salt ions but also amino acids and vitamins. Na^+^, K^+^, and Mg^2+^ were chosen as three interfering ions, which were higher in content in the culture medium (mM level). Then, 0.1 M NaCl, KCl, and MgCl_2_ were added to a series of concentration gradient CaCl_2_ standard solutions, respectively. And a series of phosphate buffer salt solutions (pH 4.2~9.2) was chosen to explore the effect of pH on working voltage.

The potentiometric selectivity coefficients of the sensor were determined according to the fixed interference method (FIM) defined by IUPAC [47]:

(1) $$K_{A,B}^{pot}=a_{A}/{\left(a_{B}\right)}^{Z_{A}/Z_{B}}$$

where $K_{A,B}^{pot}$ is the potentiometric selectivity coefficient of the primary ion A to the interfering ion B, $a_{A}$ and $a_{B}\;$ are the activity of the primary ion and the interfering ion, $Z_{A}$ and $Z_{B}$ are the charge numbers of ions.

**Figure 5:**
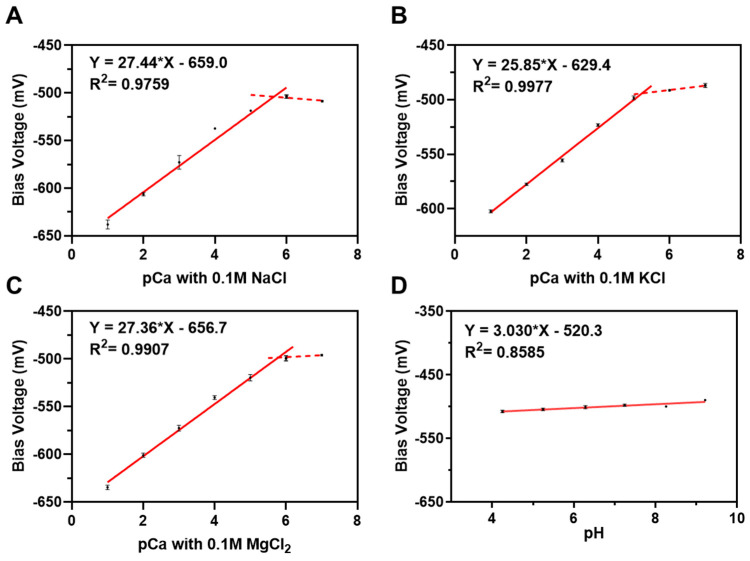
The fitting curves of calcium-ion-sensitive LAPS chips for potentiometric selectivity coefficients determination (mean ± SD, n = 3); (**A**) with 0.1 M interfering ions of Na^+^, (**B**) with 0.1 M interfering ions of K^+^, (**C**) with 0.1 M interfering ions of Mg^2+^, (**D**) with pH 4.2−9.2 solution. The solid lines represent the fitting curves within the linear working area of the sensors, and the dashed lines representfitting curves within the non-linear working area of the sensors.

The linear regression curves of bias voltage–pCa with 0.1 M Na^+^, K^+^, and Mg^2+^ interfering ions are separately shown in Figure 5A–C. The bias voltage–pH fitting curve is shown in Figure 5D. According to Equation (1), the potentiometric selectivity coefficients were calculated as follows: 3.65 for Na^+^, 3.23 for K^+^, and 4.78 for Mg^2+^. The results show that all the coefficients were greater than three. The slope of the bias voltage–pH fitting curve was almost equal to zero, indicating that the detection performance of the sensor was almost unaffected by the pH of the solution.

### 3.3. Electrophysiology Assessment of Verapamil and Bay K8644 on HL-1

**Figure 6:**
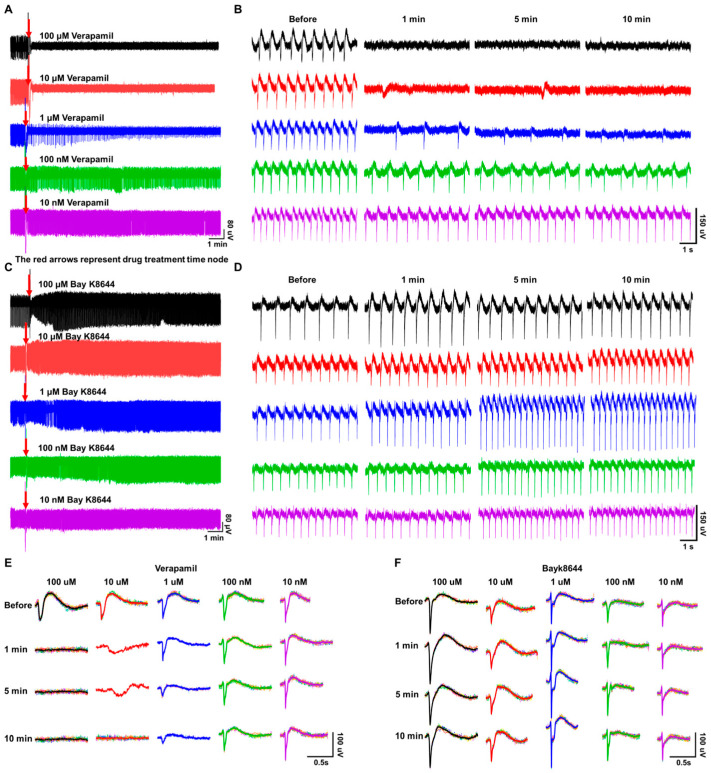
Electrophysiology results of verapamil and Bayk8644 on HL-1 cells; (**A**) overviews of HL-1 extracellular field potential (EFP) signals before and after a series of drug concentration gradient (100 μM, 10 μM, 1 μM, 100 nM, and 10 nM) verapamil treatments; (**B**) extracellular field potential signals before, then at 1 min, 5 min, and 10 min after verapamil treatment, with each signal segment lasting 10 s; (**C**) overviews of HL-1 extracellular field potential (EFP) signals before and after a series of drug concentration gradient (100 μM, 10 μM, 1 μM, 100 nM, and 10 nM) Bayk8644 treatments; (**D**) extracellular field potential signals before treatment, then at 1 min, 5 min, and 10 min after Bayk8644 treatment, with each signal segment lasting 10 s; (**E**) superposition waveform of each group of verapamil concentrations before and after 1 min, 5 min, and 10 min treatments, with a duration of one single cycle signal; (**F**) superposition waveform of each group of Bayk8644 concentrations before and after 1 min, 5 min, and 10 min treatments, with a duration of one single cycle signal.

Figure 6A,C show the extracellular field potential (EFP) signals of HL-1 cell lines before and after a series of drug concentration gradients (100 μM, 10 μM, 1 μM, 100 nM, and 10 nM) of verapamil and Bayk8644 treatment. The labels marked by the red arrow represent the drug treatment timestamps. Figure 6B,D show the extracellular field potential signals before treatment, and 1 min, 5 min, and 10 min after treatment, with each signal segment lasting 10 s. Figure 6E,F show the signal superposition waveform of each group of verapamil and Bayk8644 concentrations before treatment, and 1 min, 5 min, and 10 min after treatment, with a duration of one single cycle signal. It can be seen that after verapamil treatment, the extracellular field potential firing rate (FR) decreased in a dose-dependent manner, and the drug efficacy was time-dependent at the same concentration. It can also be observed that under high drug concentrations (100 μM and 10 μM), the cell firing function was severely inhibited and lost. We speculate that this is related to the barrier effect formed by the Ca^2+^ covering on the cell membrane blocked from entering the cell. This barrier effect competes with the Na^+^ influx, reduces the early depolarization ability of the cell membrane, and then inhibits the conduction of action potential. After the Bayk8644 treatment, the firing rate and field potential amplitude (FPA) increased.

**Figure 7:**
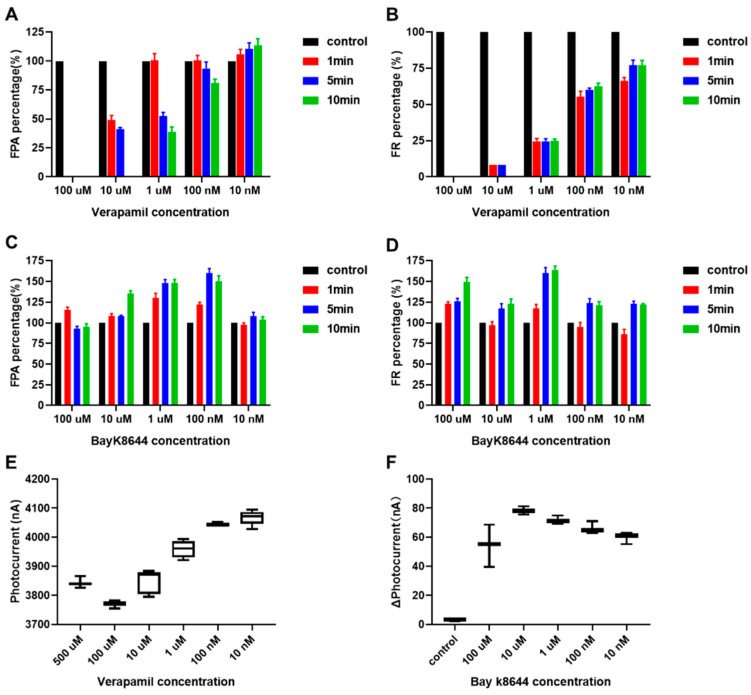
Electrophysiological statistics and calcium ion concentration statistics showing the results of drug experiments. (**A**) The normalized FPA percentage statistics results after treatment with (100 μM, 10 μM, 1 μM, 100 nM, and 10 nM) of verapamil compared to before; (**B**) the normalized FR percentage calculated statistics results after treatment with (100 μM, 10 μM, 1 μM, 100 nM, and 10 nM) of verapamil compared to before; (**C**) the normalized FPA percentage values statistics results after treatment with (100 μM, 10 μM, 1 μM, 100 nM, and 10 nM) of Bayk8644 compared to before; (**D**) the normalized FR percentage values statistics results after treatment with (100 μM, 10 μM, 1 μM, 100 nM, and 10 nM) of Bayk8644 compared to before; (**E**) photocurrent statistics results of treatment with 100 μM, 10 μM, 1 μM, 100 nM, and 10 nM of verapamil; (**F**) relative changes in photocurrent values of treatment with 100 μM, 10 μM, 1 μM, 100 nM, and 10 nM of Bayk8644.

To further quantitatively evaluate the efficacy of verapamil and Bayk8644, we selected FR and FPA as the main evaluation indicators and calculated normalized values after treatment compared to before. The results are shown in Figure 7A–D, where it can be found that both verapamil and Bayk8644 have a significant effect on the electrophysiological activity of HL-1 cardiomyocytes. As for verapamil, the FPA did not decrease, or even slightly increased, in the 1 min under low concentrations (10 nM, 100 nM, and 1 μM), and it decreased to 93.25%, 52.61%, and 41.33% in 5 min under 100 nM, 1 μM, and 10 μM, then decreased to 81.04%, 38.79%, and 0% in 10 min. Although the 100 μM group completely limited electrophysiological activity, the 10 nM group seems to have had no impact on FPA (Figure 7A). When HL-1 cardiomyocytes were treated with 10 nM, 100 nM, and 1 μM verapamil, the FR percentage decreased to 66.33%, 55.47%, and 24.53% in 1 min, and kept stable at 77.27%, 60.22% and 24.55% in 5 min. The maximum change was in the 10 μM group, in which the FPA decreased to 8.26% of the initial value, then to 0% at 10 min. The electrophysiological activity of cells in the 100 μM group was completely inhibited (Figure 7B). The effects of Bayk8644 are opposite compared to verapamil. In the 100 μM group, FPA slightly increased to 115.63% in the first minute and remained stable near the initial level (93%, 95.08%) at 5–10 min. The FPA increased to 122.24%, 130.10%, and 108.27% under 100 nM, 1 μM, and 10 μM treatment at 1 min, then increased to 160%, 148%, and 108% at 5 min, 149.92%, 148.12%, and 135.35% at 10 min. Overall, after stabilization, the 10 nM and 10 uM groups had little effect on FPA, while the 100 nM group had the strongest effect. The drug had a significant time-dependent effect on FPA, while concentration dependence was not significant (Figure 7C). Within the entire experimental concentration range, Bayk8644 has a very significant promoting effect on the FR, increasing to 123%, 124%, 160%, 117%, and 126% under 10 nM, 100 nM, 1 μM, 10 μM, and 100 μM at 5 min, and increasing to 122.06%, 121%, 163.65%, 122.91%, and 149.43% at 10 min. The 1 μM group has the most obvious effect on HL-1 cells, and the effect is positively correlated with the concentration within the range of 1 μM to 10 nM. Although Bayk8644 at high concentrations (100 μM, 10 μM) has an effect, its effect is not as good as that of the 1 μM concentration (Figure 7D). Microscopic image of HL-1 cell line cultured in T25 and MEA chips can be found in Figure S5.

### 3.4. Extracellular Calcium Ion Detection

Verapamil, one of the nondihydropyridine calcium channel blockers (CCBs), shows more myocardial preference and is more often used in patients with arrhythmia, angina, and hypertrophic cardiomyopathy (HCM) [48]. Verapamil and (-)-(S)-BayK8644 were selected as the L-type calcium channel antagonists and activators in the electrophysiology assessment experiments [49]. We verified and evaluated the short-term electrophysiological excitability changes of HL-1 cardiomyocytes under the action of verapamil and Bayk8644 through microelectrode array (MEA) chips in Section 3.3.

We did this in order to evaluate the effect of two kinds of calcium ion channel drugs more comprehensively. In addition to selecting firing frequency (FR) and field potential amplitude (FPA) as the main evaluation indicators, calcium ion concentration in the extracellular microenvironment was also detected.

Extracellular calcium ions were detected in the Claycomb medium with a series of concentration gradients (100 μM, 10 μM, 1 μM, 100 nM, and 10 nM) of verapamil and Bayk8644 as a basal state. The changes in the photocurrent values at different drug concentrations at a working voltage were detected under constant voltage mode after 1 min of treatment; the results are shown in Figure 7E,F. In Figure 7E, as the verapamil concentration decreases, the photocurrent amplitude tends to increase under this working voltage, which indicates that the concentration of calcium ions in the microenvironment decreases, which means that verapamil’s antagonism to the L-type calcium ion channel was weakened, and that calcium ions in the microenvironment can flow into cells more easily from the outside. In Figure 7F, we use the relative changes in photocurrent values before and after dosing under operating voltage to elucidate the effectiveness of Bayk8644. It can be observed that as the concentration of the Bayk8644 drug decreases, the positive change in photocurrent decreases, indicating that fewer calcium ions flow into the cell from extracellular space, supporting the effect of the drug in promoting calcium ion influx. The concentration-dependent effects of high doses of these two drugs do not meet the expected results, indicating that higher concentrations of drugs inhibit cell activity.

## 4. Conclusions

In this work, a LAPS was combined with a variety of silicone rubber membranes for the first time to establish a program-controlled multiplexed ISLAPS system for Ca^2+^ detection. The silicone-rubber ISMs had better adhesion to the silicon-based biosensors The proposed ISLAPS had good sensitivity, micromolar LOD, and good selectivity, which is the same level as, or even better than, the performance of previously reported field-effect ion-sensitive sensors. Microelectrode array (MEA) chips were employed to probe the HL-1 extracellular field potential (EFP) signal. L-type calcium channel blocker verapamil and calcium channel agonist Bayk8644 were chosen to explore the effect of ion channel drugs on extracellular calcium ion concentration in HL-1 cell lines, simultaneously. The correlation between Ca^2+^ and EFP parameters was studied to achieve a comprehensive evaluation of the efficacy of cardiovascular drugs. This platform provides more dimensional information regarding calcium ions that can be utilized for accurate drug screening.

## Data Availability

Data are contained within the article and supplementary materials.

## Supplementary Materials

The following supporting information can be downloaded at: https://www.mdpi.com/article/10.3390/bios14010016/s1, Figure S1: The response mechanism of calcium ion-sensitive LAPS; Figure S2: Schematic diagram of the method for calculating the limit of detection (LOD); Figure S3: (A) Calculation of the response time t99 for sample solution; (B) Characterization of stability in-vestigation of the sensor for a period of 180 min; Figure S4: (A) A series of photogenerated I–V characteristic curves of typical N-type LAPS under different pH conditions; (B) series of photocurrent–bias voltage (I–V) curves of the calcium-ion-sensitive LAPS chips with the corresponding concentration gradient CaCl2 solutions (10−7 M~10−1 M), ex-pressed as the average of three repeated measurements; Figure S5: (A) Microscopic image of HL-1 cell line cultured in T25; (B) Microscopic image of HL-1 cell line culture on MEA chips. Scale bar = 200 μm.
