# Supplementary material for: A Silicon-Based Field-Effect Biosensor for Drug-Induced Cardiac Extracellular Calcium Ion Change Detection
Source: Biosensors (Basel). 2023 Dec 28;14(1):16. doi: 10.3390/bios14010016 (PMC10813757; doi:10.3390/bios14010016)
Supplement: Supplementary file 1 [file biosensors-14-00016-s001.zip › biosensors-2516679-supplementary.pdf]

biosensors

Supplementary Material

A Silicon-Based Field-Effect Biosensor for Drug-Induced Cardiac Extracellular Calcium Ion Change Detection

Yong Qiu [1,2,†], Chiyu Ma [1,†], Nan Jiang [1], Deming Jiang [1], Zhengyin Yu [3], Xin Liu [1], Yuxuan Zhu [1], Weijie Yu [1], Fengheng Li [1], Hao Wan [1,2,*] and Ping Wang [1,2,3,*]

[1] Biosensor National Special Laboratory, Key Laboratory for Biomedical Engineering of Education Ministry, Department of Biomedical Engineering, Zhejiang University, Hangzhou 310027, China; zjubme_qy@zju.edu.cn (Y.Q.); mcy1996@zju.edu.cn (C.M.); 21915031@zju.edu.cn (N.J.); 21618488@zju.edu.cn (D.J.); liuxin8234@zju.edu.cn (X.L.); yuxuanzhu@zju.edu.cn (Y.Z.); yuweijie@zju.edu.cn (W.Y.); 22115006@zju.edu.cn (F.L.)
[2] The MOE Frontier Science Center for Brain Science & Brain-machine Integration, Zhejiang University, Hangzhou 310027, China
[3] State Key Laboratory of Transducer Technology, Chinese Academy of Sciences, Shanghai 200050, China; yuzy@mail.sim.ac.cn
* Correspondence: wh1816@zju.edu.cn (H.W.); cnpwang@zju.edu.cn (P.W.)
† These authors contributed equally to this work.

The response mechanism of calcium ion-sensitive LAPS was shown in Figure S1. when the electrode (coated with a calcium ISM) contacted with the analyte solution, due to the difference in calcium ion concentration between the calcium ISM and the solution, the Ca2+ in the solution will enter the calcium ISM and combine with the calcium ionophore. At the same time, due to the positive charges of Ca^{2+}, the positive charges in the membrane gradually increase. And the SiO_2 layer cannot transmit calcium ions, so positively charged calcium ions will aggregate on the side of the ISM near SiO2, that is, a positively charged layer will appear on the inner side of the ISM. According to the principle of double layer capacitance, the surface of silicon oxide near the sensitive film will induce the generation of corresponding electronic layers, thereby affecting the surface potential of the device. The following chemical reactions occurring at the interface are dynamically balanced:

$$Ca^{2+} + X \leftrightarrow CaX$$

Where X is a calcium ionophore. And the distribution of calcium ions in aqueous solutions is a random process. As the concentration of calcium ions in the analyte solution decreases, the probability of calcium ions binding to calcium ionophore in sensitive materials decreases. When the concentration is 10^{-7} M, it almost cannot cause changes in surface potential, and the sensor response at this time is background noise (see Figure 4B~D in the main manuscript).

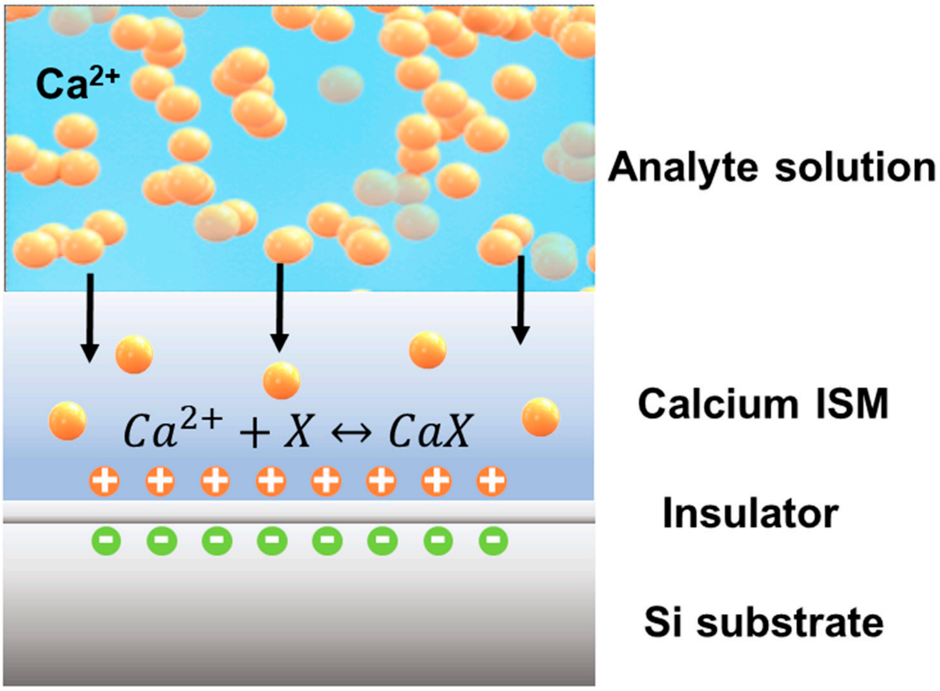

Figure S1. The response mechanism of calcium ion-sensitive LAPS.

The limit of detection (LOD) should be defined as the concentration for which, under the specified conditions, the potential E deviates from the average potential in region I by some stated arbitrary multiple of the standard error of a single measurement of the potential in region I (Figure S2). And for the sake of practical convenience, a simpler (and more convenient) definition is recommended at this time. The practical limit of detection may be taken as the activity (or concentration) at the point of intersection of the extrapolated lines as illustrated[1].

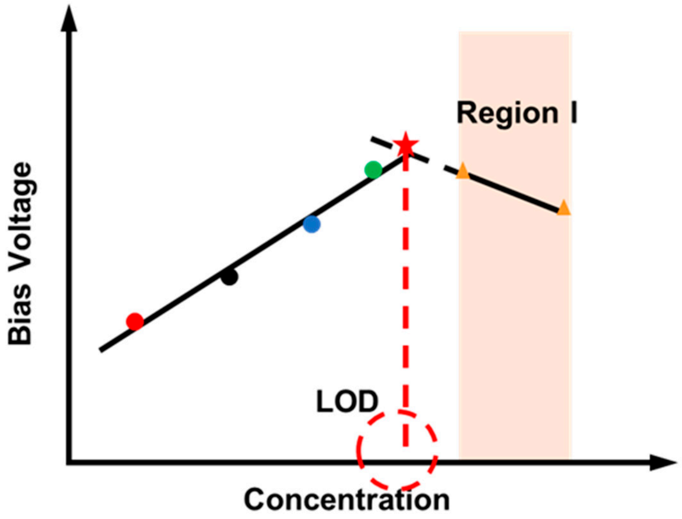

Figure S2. Schematic diagram of the method for calculating the limit of detection (LOD).

The hysteresis of response time (t99) can be seen in Figure S3A and was calculated to be 4.6 s during the constant-voltage mode measurement. And the the sensitivities of calcium ion sensitive LAPS were relatively stable for at least three months, which means the lifetime can be 3 months. Stability measurements like drift were carried out in a continuous operation by measuring the sample solution. The results showed a standard deviation of ±18.338 nA (Figure S3B) during a measurement period of 180 min.

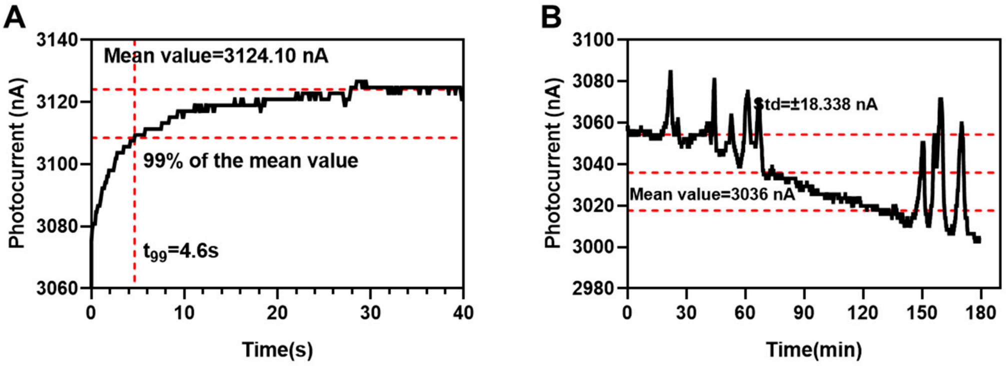

Figure S3. (**A**) Calculation of the response time t99 for sample solution; (**B**) Characterization of stability investigation of the sensor for a period of 180 min.

The characteristic curves can be divided into three regions: cutoff region, linear region, and saturation region, corresponding to the accumulation, depletion, and inversion of charge carriers in semiconductor devices, respectively (Figure S4A). When the applied bias voltage is large, the device works in the accumulation region (cutoff region), and no depletion layer is generated, resulting in almost zero photogenerated current. When the applied bias voltage decreases, a depletion layer begins to form between the semiconductor and the insulation layer, and the thickness of the depletion layer increases with the decrease of bias voltage. The photogenerated current also gradually increases, and the device operates in the depletion region (linear region). As the bias voltage further decreases, the device undergoes inversion, limited by the minority carrier (hole) concentration, the depletion layer thickness will reach its maximum, and the photogenerated current will also approach saturation, no longer following the changes in bias voltage. At this time, the device operates in the inversion region (saturation region).

An explanation for the current in the cutoff zone is not strictly zero (Red rectangular box in Figure S4B). In this study, the gate area of used LAPS chips is 1 cm^2, and the thickness of insulators is ~30 nm, which means ultrathin gate insulators on comparatively large gate areas. Because the sensitivity of potential and impedance measurements using these techniques can be greatly enhanced by reducing the thickness of the insulator[2]. However, to obtain thinner oxides the growth rate has to be lowered by reducing the growth temperature resulting in an increased number of pinholes and defects in the oxide[3]. A typical gate leakage current of 10^{-5} A/cm^2 at $V_{gate} - V_{FB} = -1$ V has been reported for 3 nm thick thermally grown oxide[2]. We supposed that the leakage current of about 0.4 μ A (this work) comes from the poor quality of SiO_2 insulation layer. Although the copolymer and P3OT to some extent inhibit the formation of aqueous layers at the electrode/ISM interface, they cannot completely eliminate it. And leakage current density of ~0.7 μ A/cm^2 can be find in Wang's work[4]. It is relatively acceptable. This issue is not the focus of this study, and future research will optimize the sensor for this leakage current.

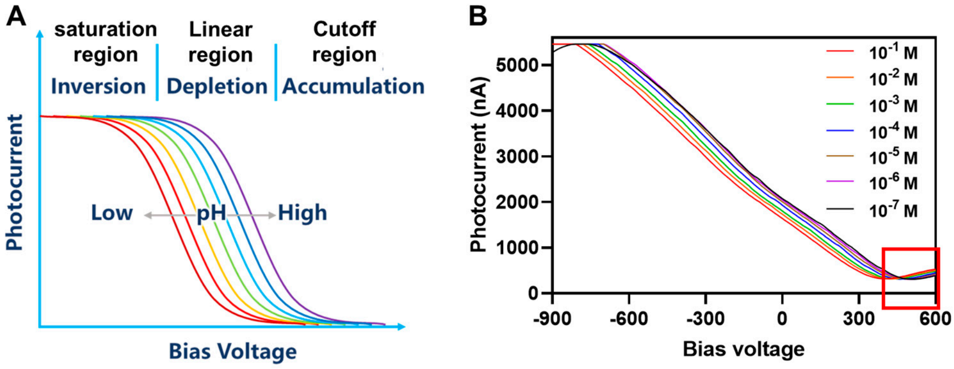

Figure S4. (**A**) A series of photogenerated I–V characteristic curves of typical N-type LAPS under different pH conditions; (**B**) series of photocurrent−bias voltage (I–V) curves of the calcium-ion-sensitive LAPS chips with the corresponding concentration gradient $CaCl_2$ solutions (10^{-7} M~10^{-1} M), expressed as the average of three repeated measurements.

We found that the firing rate and amplitude of the electrophysiological signals would basically stabilize after 1 minute of drug treatment. We selected 1 minute as the time node. Using the T25 culture flask to culture HL-1 cells (Figure S5A), when the cells fuse to 90% -100%, discard the culture medium and rinse 3 times with HBSS (without calcium and magnesium). Add 1mL of the corresponding concentration of drug then put in incubator for 1 minute. Take out the supernatant and collect it as a sample for subsequent testing. The HL-1 cardiomyocyte was cultured in T25 culture flask in a humidified incubator at 37℃ and 5% CO_2 throughout the entire experimental process. And the minimum time required to detect calcium ions concentration was approximately 5 – 10 s without sensor calibration time (Refer to Figure S3A. And we put different buffer of Ca^{2+} concentration into measurement area using the Pipette.

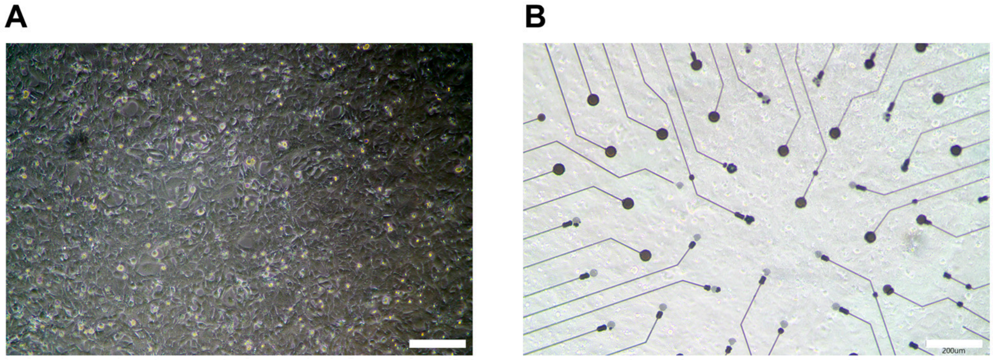

Figure S5. (**A**) Microscopic image of HL-1 cell line cultured in T25; (**B**) Microscopic image of HL-1 cell line culture on MEA chips. Scale bar = 200 μm.

References

1. Guilbault, G. Recommendations for publishing manuscripts on ion-selective electrodes. *Pure and Applied Chemistry* **1981**, *53*, 1907-1912.
2. Chen, L.; Zhou, Y.; Krause, S.; Munoz, A.G.; Kunze, J.; Schmuki, P. Repair of thin thermally grown silicon dioxide by anodic oxidation. *Electrochimica acta* **2008**, *53*, 3395-3402.
3. Marathe, V.G.; Paily, R.; DasGupta, A.; DasGupta, N. A model to study the effect of selective anodic oxidation on ultrathin gate oxides. *IEEE transactions on electron devices* **2004**, *52*, 118-121.
4. Wang, J.; Zhou, Y.; Watkinson, M.; Gautrot, J.; Krause, S. High-sensitivity light-addressable potentiometric sensors using silicon on sapphire functionalized with self-assembled organic monolayers. *Sensors and Actuators B: Chemical* **2015**, *209*, 230-236.
